# The Sweetpotato BTB-TAZ Protein Gene, *IbBT4*, Enhances Drought Tolerance in Transgenic *Arabidopsis*

**DOI:** 10.3389/fpls.2020.00877

**Published:** 2020-06-23

**Authors:** Yuanyuan Zhou, Hong Zhai, Shaozhen He, Hong Zhu, Shaopei Gao, Shihan Xing, Zihao Wei, Ning Zhao, Qingchang Liu

**Affiliations:** ^1^Key Laboratory of Sweetpotato Biology and Biotechnology, Ministry of Agriculture and Rural Affairs/Beijing Key Laboratory of Crop Genetic Improvement/Laboratory of Crop Heterosis and Utilization, Ministry of Education, College of Agronomy & Biotechnology, China Agricultural University, Beijing, China; ^2^College of Agronomy, Qingdao Agricultural University, Qingdao, China

**Keywords:** sweetpotato, *IbBT4*, drought tolerance, BR signaling pathway, *Arabidopsis*

## Abstract

BTB-TAZ (BT)-domain proteins regulate plant development and pathogen defense. However, their roles in resistance to abiotic stresses remain largely unknown. In this study, we found that the sweetpotato BT protein-encoding gene *IbBT4* significantly enhanced the drought tolerance of *Arabidopsis*. *IbBT4* expression was induced by PEG6000, H_2_O_2_ and brassinosteroids (BRs). The *IbBT4*-overexpressing *Arabidopsis* seeds presented higher germination rates and longer roots in comparison with those of WT under 200 mM mannitol stress. Under drought stress the transgenic *Arabidopsis* plants exhibited significantly increased survival rates and BR and proline contents and decreased water loss rates, MDA content and reactive oxygen species (ROS) levels. *IbBT4* overexpression upregulated the BR signaling pathway and proline biosynthesis genes and activated the ROS-scavenging system under drought stress. Yeast two-hybrid (Y2H) and bimolecular fluorescence complementation (BiFC) assays revealed that the IbBT4 protein interacts with BR-ENHANCED EXPRESSION 2 (BEE2). Taken together, these results indicate that the *IbBT4* gene provides drought tolerance by enhancing both the BR signaling pathway and proline biosynthesis and further activating the ROS-scavenging system in transgenic *Arabidopsis*.

## Introduction

The effects of drought on agricultural production are increasing worldwide (Zhu, 2002; Yang et al., 2010; Boyer et al., 2013; Chiogna et al., 2018). Phytohormones are essential for plant growth, development and protective responses against severe environmental stresses (Dong et al., 2014; Vargas et al., 2014; Jin et al., 2016). Brassinosteroids (BRs) compose a group of naturally- occurring steroidal phytohormones that are involved in the regulation of adaptation to environmental stresses, such as salinity, drought, heat and cold, as well as multiple aspects of growth and development in plants (Nolan et al., 2017; Planas-Riverola et al., 2019).

In the BR biosynthesis pathway, the BR-specific biosynthetic precursor campesterol (CR) is first converted to campestanol (CN) and then to brassinolide (BL) via the early and late C-6 oxidation pathways. In addition, early C-22 and C-23 hydroxylation branches are the dominant upstream BR biosynthesis pathways (Ohnishi et al., 2006a, b). Several key enzymes, including DWARF4 (DWF4), constitutive photomorphogenesis and dwarfism (CPD), De-etiolated-2(DET2), C-6 oxidase 1, 2 (BR6ox1, 2), and rotundifolia 3 (ROT3), regulate BR biosynthesis (Kim et al., 1999; Nomura et al., 2001; Shimada et al., 2001; Ohnishi et al., 2012). The BR signaling pathway starts with the membrane-localized receptor brassinosteroid-insensitive 1 (BRI1) and co-receptor BRI1-associated kinase 1 (BAK1) (Li and Nam, 2002; Kinoshita et al., 2005). Via inactivation of brassinosteroid-insensitive 2 (BIN2), a negative regulator of BR signaling, BR signaling ultimately culminates in the activation of BRI1-EMS suppressor 1 (BES1)/brassinazole-resistant 1 (BZR1) family transcription factors (Wang et al., 2002; Yin et al., 2002). Thus, the transcription factors BZR1 and BES1 directly regulate the transcription of BR-responsive genes in plants (Wang et al., 2002; Nolan et al., 2017; Planas-Riverola et al., 2019).

The plant-specific BTB-TAZ (BT)-domain protein subfamily, including five members, BT1 to BT5, belong to the bric-a-brac/tramtrack/broad complex (BTB) protein family. These members contain an N-terminal BTB domain, a transcriptional adapter zinc finger (TAZ) domain and a C-terminal calmodulin-binding (CaMB) domain (Du and Poovaiah, 2004). Ren et al. (2007) found that AtBT2 interacts with AtTAC1 and regulates primary root growth in *Arabidopsis*. In addition, AtBT2 and AtBT3 were found to be essential for gametophyte development in *Arabidopsis* (Robert et al., 2009). By interacting with AtGTE9 and AtGTE11, AtBT2 imparted resistance to abscisic acid (ABA) and glucose in *Arabidopsis* (Mandadi et al., 2009; Misra et al., 2018). Overexpression of *AtBT2* partially rescued growth inhibition caused by reductions in AtNLP activity and affected the development of nitrate-dependent lateral roots in *Arabidopsis* (Sato et al., 2017). MdBT2 delayed leaf senescence by inducing the ubiquitination and degradation of MdbHLH93 in transgenic *Arabidopsis* and apple (An et al., 2019). *AtBT4* was reported to regulate resistance to *Botrytis cinerea* and *Pseudomonas syringae* in *Arabidopsis* (Hao et al., 2013; Zheng et al., 2019). However, the roles of BTs in the resistance to abiotic stresses remain largely unknown in plants.

In this study, we cloned a BTB-TAZ domain protein-encoding gene *IbBT4* from sweetpotato (*Ipomoea batatas* (L.) Lam.). It was found that this gene provided drought tolerance by enhancing both the BR signaling pathway and proline biosynthesis and further activating the ROS-scavenging system in transgenic *Arabidopsis*.

## Materials and Methods

### Plant Materials

The sweetpotato line Xushu55-2, which is tolerant to drought, was used for isolating the *IbBT4* gene and analysing its expression profile. The plants were cultured on Murashige and Skoog (MS) media at 27 ± 1 °C under 13 h of daylight and 54 μM m^–2^ s^–1^. *Nicotiana benthamiana* was used for the subcellular localization of the IbBT4 protein. The function of this gene was analyzed in *Arabidopsis thaliana* Columbia-0 (WT). *N. benthamiana* and *A. thaliana* plants were grown in an artificial climate chamber at 22 ± 1°C and 30% humidity under 16 h of daylight and 54 μM m^–2^s^–1^.

### Cloning and Sequence Analysis of *IbBT4* and Its Promoter

Total complete RNA was isolated from *in vitro*-grown Xushu55-2 plants and then reverse transcribed to generate cDNA (Zhou et al., 2019). Based on the expressed sequence tag (EST) selected from the transcriptome sequencing data of Xushu55-2 (Zhu et al., 2018), the 5′-untranslated region (UTR) and 3′-UTR of *IbBT4* were amplified using rapid amplification of cDNA ends (RACE) procedure using 5′- Full RACE Kit and 3′-Full RACE Core Set Ver.2.0 Kit (TaKaRa, Beijing, China). The cDNA sequence was analyzed by NCBI^1^. The coding sequence (CDS) was cloned by PCR with specific primers. Its genomic sequence was cloned from Xushu55-2 genomic DNA via PCR in conjunction with specific primers. The promoter region was cloned with Universal Genome Walker 2.0 Kit (TaKaRa, Dalian, China). All of the specific primers are listed in Supplementary Table S1. *IbBT4* was annotated in the NCBI databases^2^. Amino acid sequence alignments, phylogenetic relationships and exon-intron structure were analyzed with DNAMAN software, MEGA 7.0 software and the Splign tool, respectively, and the molecular weight and theoretical isoelectric point (*p*I) were calculated via online software (Kang et al., 2019). The *cis*-acting regulatory elements in the promoter region were analyzed via the PlantCARE database.

### Subcellular Localization

The coding sequence of *IbBT4* was amplified with specific primers (Supplementary Table S1) and inserted into the pMDC83 vector together with green fluorescent protein (GFP). The fusion construct (35S:*IbBT4:GFP*) and the empty vector (*35S:GFP*, control) were subsequently transferred into *Agrobacterium tumefaciens* strain EHA105, respectively, which was then infiltrated into the leaf epidermal cells of 4-week-old *N. benthamiana* plants with a needleless syringe. At 48 h after agroinfiltration, the infiltrated leaf sections were observed at room temperature using a laser scanning confocal microscope with an argon laser (Olympus, Tokyo, Japan). The cells containing GFP were excited at 488 nm and emissions were recorded at 505–555 nm.

### Expression Analysis of *IbBT4*

Total RNA from the root, stem and leaf tissues of the *in vitro*-grown Xushu55-2 plants and the leaf, stem, hair root, fibrous root and storage root tissues of 80 days old field-grown plants was extracted for analysing the expression of *IbBT4* using quantitative real-time PCR (qRT-PCR), and the specific primers used are listed in Supplementary Table S1 (Zhou et al., 2019). The expression levels of *IbBT4* in different tissues were normalized to those of *Ibactin* (AY905538), and the relative expression levels were calibrated using leaf tissue. Four-week-old *in vitro*-grown plants of Xushu55-2 were treated with Hoagland solution that comprised H_2_O (control), 30% PEG6000, 100 μM H_2_O_2_ and 100 nM BR, respectively, and after 0, 1, 3, 6, 12, and 24 h of treatment, whole plants were sampled for analysing the *IbBT4* expression profiles. The expression levels of *IbBT4* in the different treatments were normalized to those of *Ibactin* (AY905538) and calibrated using the plants sampled at 0 h after treatment. Three plants were used per treatment, and 3 biological replications were included in each treatment.

### *Arabidopsis* Transformation

The coding sequence of *IbBT4* was inserted into a pCAMBIA1300 vector to generate a pCAMBIA1300-*35S-IbBT4* overexpression vector construct using specific primers (Supplementary Table S1), after which the construct was then introduced into *A. tumefaciens* strain GV3101. The floral-dip method was used to produce transgenic *Arabidopsis* plants, which were subsequently grown in pots to produce T_3_ seeds by screening with 50 mg/L hygromycin. The expression of *IbBT4* in the transgenic *Arabidopsis* plants was analyzed according to the method of Zhou et al. (2019).

### Drought Tolerance Assay of Transgenic *Arabidopsis*

To identify the drought tolerance of transgenic *Arabidopsis*, T_3_ and wild-type (WT) seeds were sown in the same plates with 1/2- strength MS media with or without (control) 200 mM mannitol, and after 5 days, their germination rates were measured. Approximately 50 seeds of each line were investigated. Five-day-old seedlings were cultured on 1/2 MS media with or without (control) 200 mM mannitol and after 2 weeks the primary root length was measured. Each material was treated with 5 seedlings per treatment and 3 biological replications were included. Furthermore, one-week-old seedlings growing on 1/2 MS media were planted in pots (7 cm × 7 cm) filled with a soil:vermiculite:humus mixture (1:1:1, v/v/v) for 2 weeks and then stressed by a 4-week-long drought followed by 2-days of re-watering to observe the phenotypes; the seedlings were grown in an artificial climate chamber at 22 ± 1°C and 30% humidity under 16 h of daylight and 54 μM m^–2^ s^–1^. Plants grown in pots under normal conditions for 6 weeks were used as controls. The experiments were set up with 3 biological replications and 5 plants per treatment.

### Measurements of Components and Expression Analysis of Genes Related to Drought Tolerance

The leaves were detached from the T_3_ and WT plants grown for 4 weeks under normal conditions and their water loss rates were analyzed as described by Li et al. (2019). The leaves of the T_3_ and WT plants grown for 2 weeks under normal conditions followed by 2 weeks of drought stress and for 4 weeks under normal conditions (control) were used to measure the proline and MDA contents and SOD activity with assay kits (Comin Biotechnology Co., Ltd., Suzhou, China). Their BR and reactive oxygen species (ROS) contents were determined using an indirect enzyme-linked immune sorbent assay (ELISA) kit (Comin Biotechnology Co., Ltd., Suzhou, China) and nitro blue tetrazolium (NBT) staining (Song et al., 2015), respectively. The leaves of these plants were also used to analyze the transcript levels of the genes involved in the BR biosynthesis and signaling pathways, proline biosynthesis and the ROS-scavenging system using qRT-PCR and *Atactin* (NM112764); the with specific primers used are listed in Supplementary Table S1. The relative expression levels in T_3_ were calibrated using those of WT. Three plants were evaluated per sample, and 3 biological replications were included.

### Yeast Two-Hybrid (Y2H) Assays

The coding sequence of *IbBT4* was fused to the yeast expression vector pGBKT7 (pBD) for transactivation activity assays (Zhou et al., 2019). The pBD-*IbBT4* bait plasmid and prey plasmid library components were co-transformed into Y2H Gold yeast according to the Matchmaker^TM^ Gold Yeast Two-Hybrid System User Manual protocol (Clontech). The coding sequences of *IbBEE2* and *AtBEE2* were cloned into a pGADT7 (pAD) vector, after which the bait and prey plasmids were transformed into Y2H Gold yeast. The pAD/pBD-*IbBT4* and pBD/pAD-*BEE2* were used as negative controls and the pBD-53/pAD was used as a positive control. The transformed yeasts were spread on SD/-Trp/-His/-Leu/-Ade/X-a-Gal plates to test protein-protein interactions at 30°C for 5–8 days. The specific primers used are shown in Supplementary Table S1.

### Bimolecular Fluorescence Complementation (BiFC) Assays

The coding sequence of *IbBT4* was inserted into pSPYNE-35S, which was then fused to the N-terminus of yellow fluorescence protein (YFP); moreover, the coding sequence of *IbBEE2* or *AtBEE2* was inserted into pSPYCE-35S, which was then fused to the C-terminus of YFP (Supplementary Table S1). The plasmids were introduced into *A. tumefaciens* strain GV3101, the cells of which were then infiltrated into *N. benthamiana* leaves. After 2 days, YFP signals were observed under a confocal laser scanning microscope (FV-1000, Olympus, Japan) equipped with an argon laser. The excitation wavelength was 488 nm and recording wavelength ranged from 505 to 555 nm.

### Statistical Analysis

The data are presented as the means ± SEs of three biological replicates and were analyzed with Student’s *t*-tests (two-tailed analyses). Significance levels at *P* < 0.05 and *P* < 0.01 are indicated with ^∗^ and ^∗∗^, respectively.

## Results

### Sequence Analysis of *IbBT4* and Its Promoter

**FIGURE 1:**
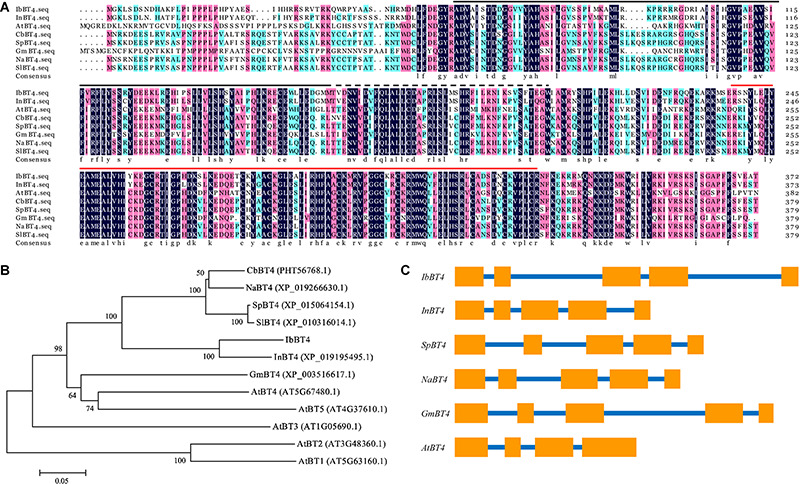
Sequence analysis of IbBT4. **(A)** Sequence alignment of IbBT4 and its homologs from other plant species. The conserved amino acids are indicated by a dark background. 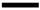, BTB_POZ_BT domain; 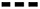, ZnF_TAZ domain; 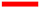, BACK domain. **(B)** Phylogenetic analysis of IbBT4 and BT proteins from other plant species. **(C)** Exon and intron constituents of *BT4* genes. The exons are represented by boxes; the introns, by lines.

The *IbBT4* gene was cloned from the drought-tolerant sweetpotato line Xushu55-2 by RACE and submitted to GenBank (accession no. MT387197). The 1792 bp cDNA of *IbBT4* contains a 323 bp 5′-UTR and a 350 bp 3′-UTR. Its CDS was 1119 bp and encoded for a polypeptide of 372 aa with a molecular weight of 43.13 kDa and a predicted *p*I of 9.46. This gene contains a BTB_POZ_BT domain, a ZnF_TAZ domain and a BACK domain (Figure 1A). The IbBT4 protein was most closely related to that of *Ipomoea nil* (XP_019195495.1, 86%) and shared high sequence identity with BT4 proteins from *Capsicum baccatum* (PHT56768.1, 68.38%), *Solanum pennellii* (XP_015064154.1, 67.52%), *Nicotiana attenuata* (XP_019266630.1, 68.41%), *Glycine max* (XP_003516617.1, 52.08%), and *Arabidopsis thaliana* (AT5G67480.2, 49.48%) (Figure 1B). The 2804 bp genomic DNA of *IbBT4* consisted of 5 exons and 4 introns, similar to that of *InBT4*, *CbBT4*, *SpBT4*, *NaBT4*, and *GmBT4*, but not *AtBT4* (Figure 1C). The 1910 bp promoter region of *IbBT4* contains several stress-responsive *cis*-acting regulatory elements, including GARE- motif, O2-site, LTR, TC-rich repeats, TCA-element, MBS, ARE, and MBSI (Supplementary Figure S1). The presence of these *cis*-acting elements in the promoter regions suggests that the expression of *IbBT4* might be induced by abiotic stresses.

### Subcellular Location of IbBT4

**FIGURE 2:**
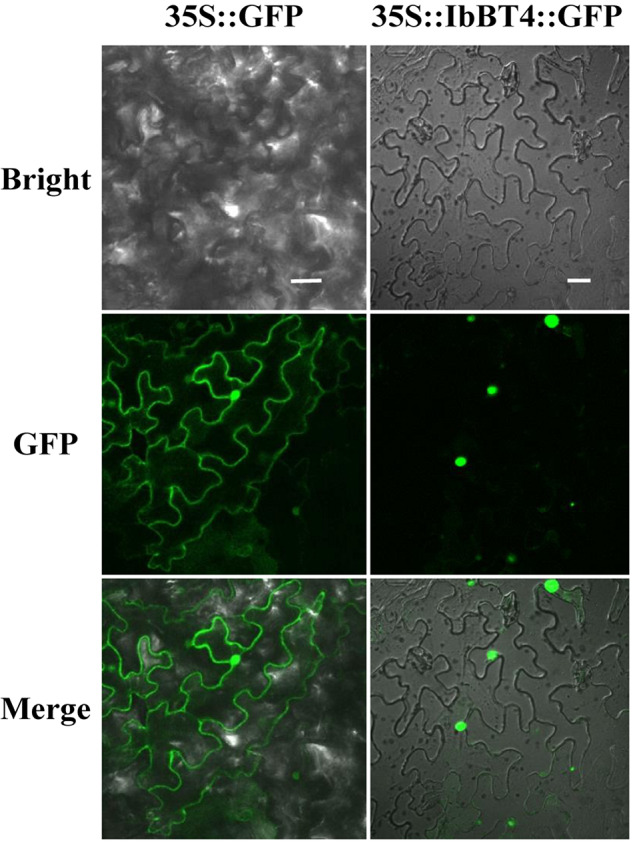
Subcellular localization of IbBT4 in tobacco leaf hypodermal cells. Confocal scanning microscopy images showing the localization of IbBT4-GFP fusion proteins to nuclei in the right column vs. GFP as the control in the left column. Bars = 20 μm.

To analyze the protein subcellular localization, the CDS of *IbBT4* (no stop codon) was fused with GFP and transiently expressed in the leaf epidermal cells of *N. benthamiana*. The images from the leaf epidermal cells showed IbBT4-GFP fluorescence in the nuclei, while GFP fluorescence of the control was observed in the entire cell (Figure 2). Thus, IbBT4 was localized in the nucleus.

### Expression Profiles of *IbBT4* in Sweetpotato

**FIGURE 3:**
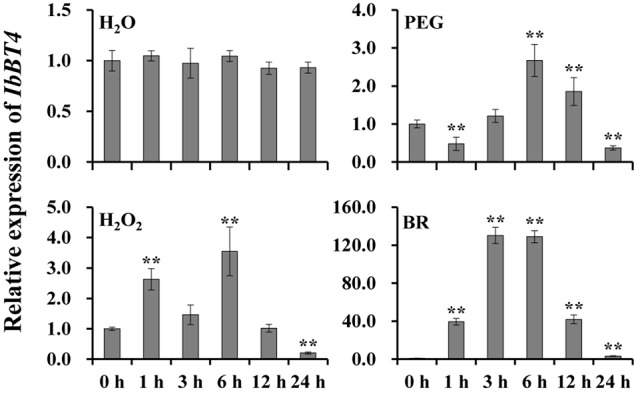
Expression analysis of *IbBT4* in *in vitro*-grown Xushu55-2 plants after different time points (h) in response to H_2_O (control), 30% PEG6000, 100 μM H_2_O_2_ and 100 nM BR, respectively. The data are presented as the means ± SEs (*n* = 3). **Indicates a significant difference at *P* < 0.01 according to Student’s *t*-test.

To investigate the potential working site of *IbBT4* in sweetpotato, we analyzed its expression level in different tissues of Xushu55-2. The highest expression level was found in the leaves (Supplementary Figure S2). To further analyze its potential function in response to abiotic stresses, the expression of *IbBT4* was checked using the 4-week-old *in vitro*-grown plants of Xushu55-2. It was found that *IbBT4* was strongly induced by PEG6000, H_2_O_2_ and BR, and peaked (2.67-fold) at 6 h, (3.54-fold) at 6 h, and (130.18-fold) at 3 h (Figure 3). These results indicate that *IbBT4* might be involved in drought, H_2_O_2_ and BR response pathways.

### Enhanced Drought Tolerance of Transgenic *Arabidopsis*

To identify the function of *IbBT4*, this gene was transferred to *Arabidopsis*. Seventeen putatively transgenic *Arabidopsis* plants were produced and 8 of them, named L1, L2, …, L8, were confirmed to be transgenic by PCR analysis. T_3_ lines were generated from these 8 transgenic plants by screening with hygromycin. qRT-PCR analysis indicated that 3 (L2, L3, and L7) of them had relatively high expression levels of *IbBT4* (Supplementary Figure S3). These 3 transgenic lines were selected, and their drought tolerance was evaluated.

**FIGURE 4:**
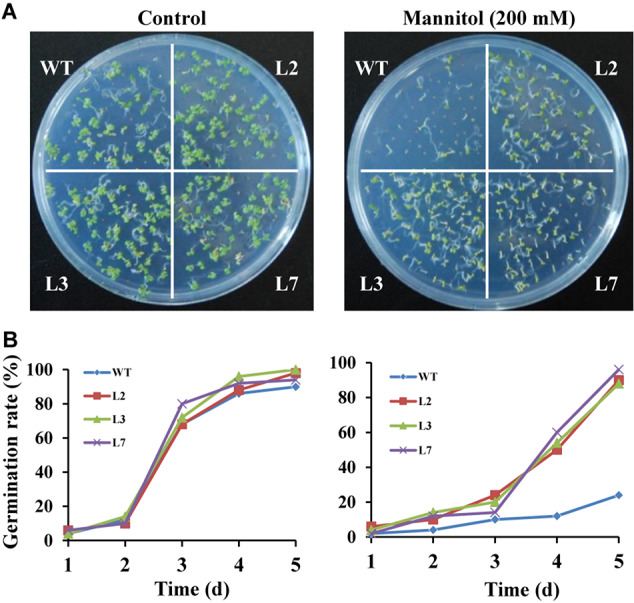
Germination assays of transgenic *Arabidopsis* and WT seeds sown for 5 days on 1/2 MS media without (control) or with 200 mM mannitol. **(A)** Growth vigor of the transgenic and WT seedlings. **(B)** Germination rates of the transgenic and WT seeds.

**FIGURE 5:**
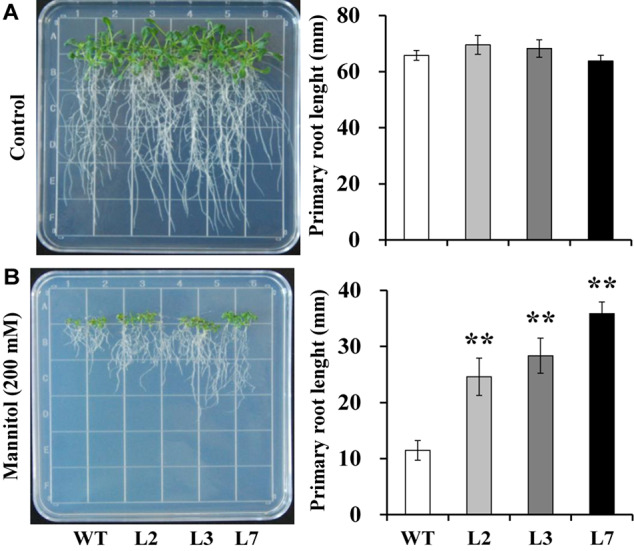
Responses of transgenic *Arabidopsis* and WT seedlings cultured for 2 weeks on 1/2 MS media without (control, **A**) or with 200 mM mannitol **(B)**. The data are presented as the mean ± SEs (*n* = 3). **Indicates a significant difference from that of WT at *P* < 0.01 by Student’s *t*-test.

**FIGURE 6:**
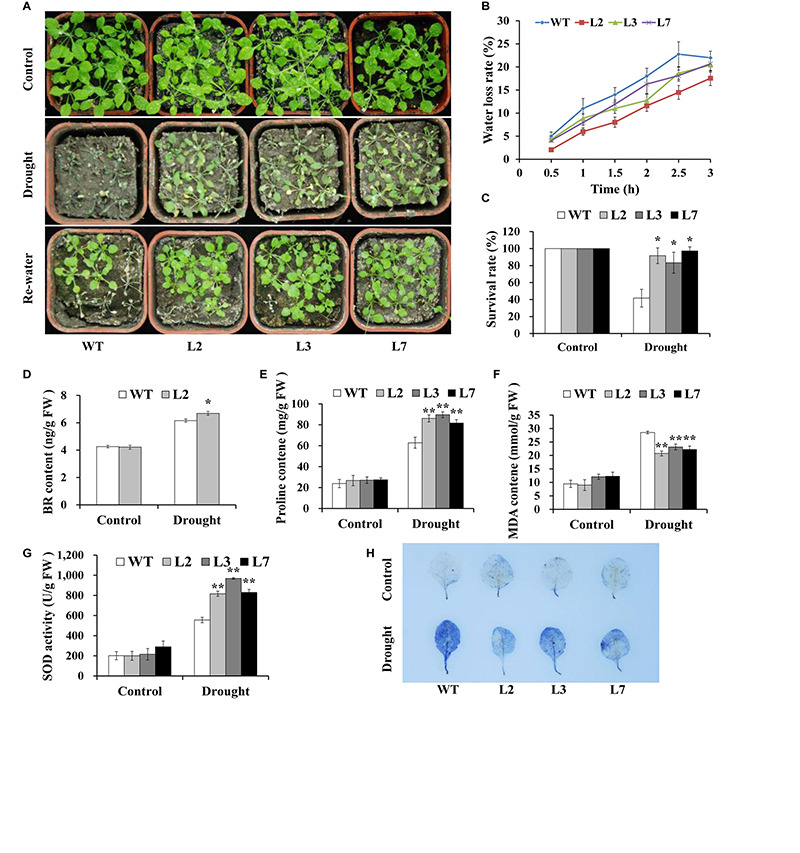
Responses of transgenic *Arabidopsis* and WT plants to drought stress. **(A)** Phenotypes of the transgenic plants vs. WT stressed by a 4-week-long drought period followed by 2 days of re-watering after 2 weeks of normal treatment. A 6-week-long normal treatment was used as a control. **(B)** Water loss rate of detached leaves from transgenic *Arabidopsis* and WT plants grown for 4 weeks under normal conditions. **(C)** Survival rate of transgenic and WT plants after 2 days of re-watering. **(D–G)** BR, proline and MDA contents and the SOD activity in the leaves of the transgenic and WT plants grown for 2 weeks under normal conditions followed by 2 weeks of drought stress and for 4 weeks under normal conditions (control), respectively. **(H)** NBT staining. * and ** indicate significant differences from those of WT at *P* < 0.05 and *P* < 0.01, respectively, according to Student’s *t*-test.

To investigate whether *IbBT4* provides plant drought tolerance, transgenic *Arabidopsis* plants were evaluated *in vitro* and *in vivo* for their drought tolerance. No differences in seed germination rates between the transgenic lines and WT were observed under normal conditions, but all of the transgenic lines showed significantly higher germination rates than did the WT under 200 mM mannitol treatment (Figures 4A,B). Five-day-old transgenic and WT seedlings were used for drought stress tests. The seedlings were grown on 1/2 MS media with or without 200 mM mannitol for 2 weeks. The primary root length was measured as an indicator of the stress tolerance of plants. Similar growth between the transgenic lines and WT was observed on 1/2 MS media without mannitol, but the transgenic plants produced significantly longer primary roots than did the WT on 1/2 MS media with 200 mM mannitol (Figures 5A,B). To further evaluate the drought tolerance of the transgenic lines, one-week-old seedlings grown on 1/2 MS media were potted for 2 weeks and then stressed by a 4-week drought in an artificial climate chamber. Under normal conditions, the transgenic lines and WT exhibited similar phenotypes (Figure 6A), but lower water loss rates were found in the transgenic lines than in the WT (Figure 6B). Under drought stress, compared with WT, the transgenic lines showed better growth, increased survival rates, increased BR and proline contents and increased SOD activity but decreased MDA content and decreased ROS accumulation (Figures 6A–H). Taken together, these results demonstrated that overexpression of *IbBT4* enhanced drought tolerance in *Arabidopsis.*

### Underlying Mechanism by Which *IbBT4* Provides Drought Tolerance

**FIGURE 7:**
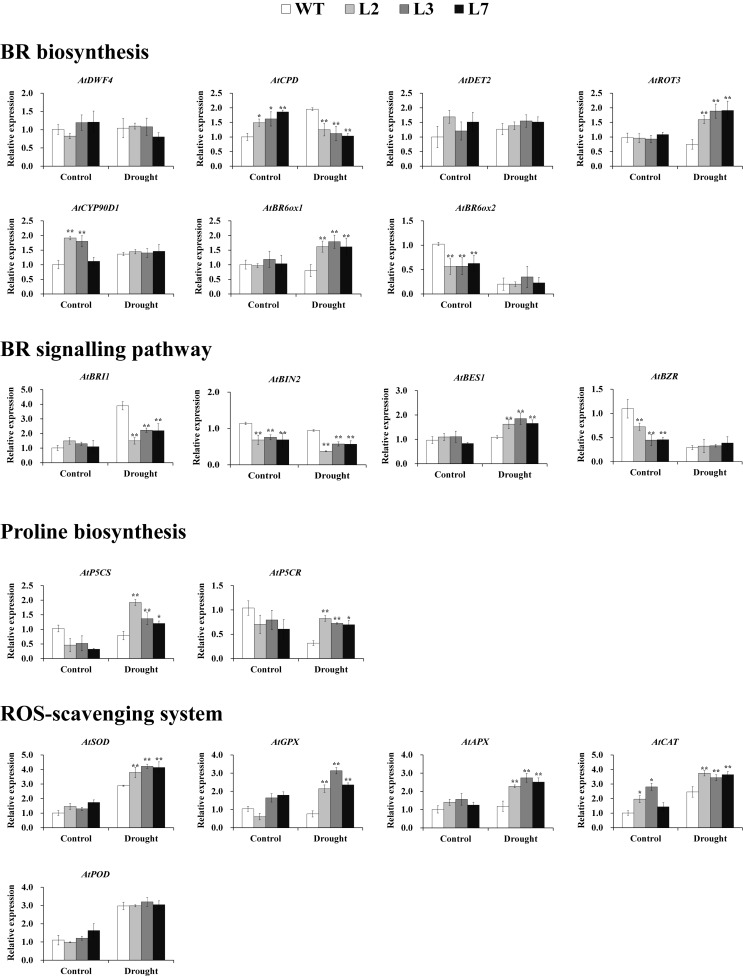
Transcript levels of relevant genes in the leaves of transgenic *Arabidopsis* and WT plants grown for 2 weeks under normal conditions followed by 2 weeks of drought stress and for 4 weeks under normal conditions (control), respectively. * and ** indicate significant differences from those of WT at *P* < 0.05 and *P* < 0.01, respectively, according to Student’s *t*-test.

To explore the underlying mechanism of the involvement of *IbBT4* in drought tolerance, the genes involved in the BR signaling pathway, proline biosynthesis and the ROS-scavenging system were analyzed by qRT-PCR using the leaves of the T_3_ and WT plants grown for 2 weeks under normal conditions followed by 2-week-long drought stress and for 4 weeks under normal conditions. Under drought stress, the genes involved in BR biosynthesis showed different expression patterns. *AtROT3* and *AtBR6ox1* were upregulated, and *AtCPD* was downregulated in the transgenic *Arabidopsis* lines compared with the WT; *AtDWF4*, *AtDET2*, *AtCYP90D1*, and *AtBR6ox2* showed no differences (Figure 7). In the BR signaling pathway, *AtBES1* was upregulated, *AtBIN* and *AtBRI1* were downregulated, and *AtBZR1* exhibited no differences under drought stress (Figure 7). Two key enzyme genes, *AtP5CR* and *AtP5CS*, which are involved in proline biosynthesis were upregulated under drought stress (Figure 7). The ROS-scavenging genes encoding superoxide dismutase (SOD), glutathione peroxidase (GPX), peroxidase (APX) and catalase (CAT), but not peroxidase (POD), were systematically upregulated under drought stress (Figure 7).

**FIGURE 8:**
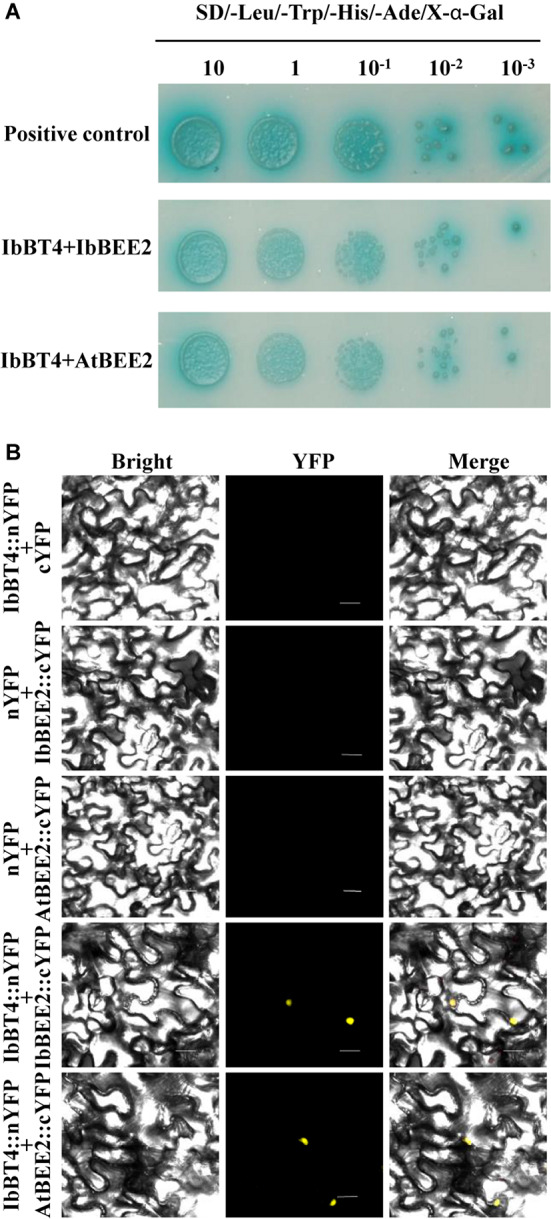
IbBT4 interacts with BEE2 in the nucleus. **(A)** Y2H assay revealing that IbBT4 interacts with BEE2 in Y2HGold cells. **(B)** BiFC assay showing that IbBT4 interacts with BEE2 in the tobacco nucleus. The yellow fluorescent protein (YFP) signals were predominantly localized in the nucleus.

The yeast cells harboring pGAL4 (+) grew well and turned blue, but the cells carrying pBD-*IbBT4* and pBD (−) failed to grow on media containing X-α-Gal (SD/-Trp/-His/X), indicating that IbBT4 had no transactivation activity (Supplementary Figure S4). To further explore the functional mechanism of IbBT4, we performed Y2H assays. The yeast cells carrying negative controls failed to grow on media containing X-α-Gal (SD/-Leu/-Trp/-His/-Ade), and the positive control as well as yeast cells with pBD-BT4/pBEE2 were grew well and turned blue. The results showed that IbBT4 interacted with IbBEE2/AtBEE2 in cells of the yeast strain Y2H Gold (Figure 8A). The BiFC assay also showed that IbBT4 interacted with IbBEE2/AtBEE2 in the tobacco nucleus (Figure 8B). Friedrichsen et al. (2002) found that BEE2 positively regulated BR signaling in *Arabidopsis*. Therefore, it is inferred that IbBT4 might enhance drought tolerance by interacting with AtBEE2 in transgenic *Arabidopsis*.

## Discussion

### *IbBT4* Enhances Drought Tolerance

The BTs have been reported to regulate plant development and pathogen defense (Du and Poovaiah, 2004; Ren et al., 2007; Robert et al., 2009; Sato et al., 2017; Zheng et al., 2019). However, their roles in the regulation of responses to abiotic stresses are still unclear. In our study, we cloned the *IbBT4* gene from sweetpotato and found that the IbBT4 protein shared the highest identity with that of AtBT4 from *Arabidopsis* (Figure 1B). In *Arabidopsis*, BT1 and BT2 are localized in the nucleus and the cytoplasm, whereas BT3, BT4 and BT5 were determined to be as cytosolic proteins (Robert et al., 2009). This study showed that IbBT4 was localized in the nucleus (Figure 2). Thus, it is possible that the function of IbBT4 is functionally homologous to that of AtBT1 and AtBT2. The expression of *IbBT4* was induced by PEG6000, H_2_O_2_ and BR (Figure 3) and compared with the WT plants, the *IbBT4*-overexpressing *Arabidopsis* plants displayed significantly enhanced drought tolerance (Figures 4–6). These results suggest that *IbBT4* positively regulates plant drought tolerance.

### *IbBT4* Positively Regulates the BR Signaling Pathway

BRs regulate multiple aspects of plant growth and adaptations to cope with abiotic stresses, such as drought, cold, heat, and salinity stress (Zhu et al., 2013; Singh and Savaldi-Goldstein, 2015; Nolan et al., 2017; Planas-Riverola et al., 2019). The *Arabidopsis* BR biosynthetic gene *AtDWARF4* conferred drought tolerance to *Brassica napus* (Sahni et al., 2016). Morevoer, the *Arabidopsis* BR-deficient mutant *det2*-*9* accumulates relatively high levels of O_2_^–^ in the roots (Lv et al., 2018). In the BR signaling pathway, *AtBRI1* positively regulates *Arabidopsis* responses to cold and drought stresses (Kim et al., 2010; Ye et al., 2017). As a repressor of BR signaling, BIN2 modulates the degradation of BES1 (Nolan et al., 2017). Gain-of-function *Arabidopsis* BR mutants (*bes1-D*) showed decreased drought tolerance (Ye et al., 2017), and overexpression of *TaBZR2* led to enhanced drought tolerance in wheat (Cui et al., 2019).

**FIGURE 9:**
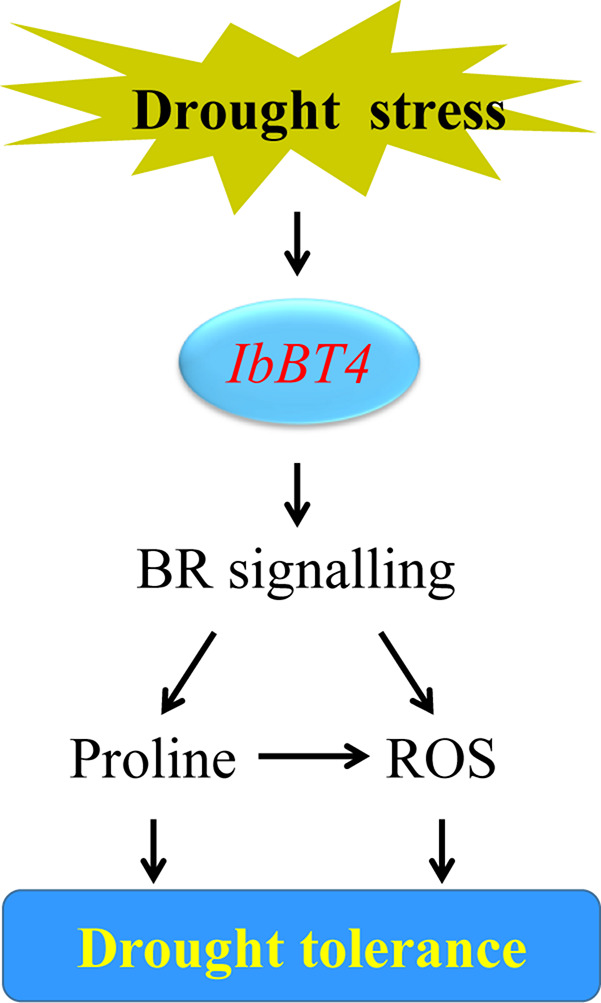
Diagram showing a proposed model for the regulation of IbBT4 in the drought tolerance of transgenic *Arabidopsis*.

In our study, an increased BR level was detected in the *IbBT4*-overexpressing *Arabidopsis* plants under drought stress (Figure 6D). Overexpression of *IbBT4* also led to upregulation of *AtROT3*, *AtBR6ox1* and *AtBES1* and downregulation of *AtBIN2* under drought stress (Figure 7). These results indicated that *IbBT4* positively regulates the BR signaling pathway in transgenic *Arabidopsis* (Figure 9). Furthermore, we found that IbBT4 interacts with BEE2 through Y2H and BiFC assays (Figure 8). Friedrichsen et al. (2002) identified three closely related basic helix-loop-helix (bHLH) transcription factors, BEE1, BEE2, and BEE3, as products of early response genes required for the full BR response and suggested that they are functionally redundant positive regulators of BR signaling in *Arabidopsis*. Similarly, Zhao et al. (2016) showed that MdBT1 and MdBT2 from apple interact with MdbHLH104, and MdBT2 regulates MdbHLH104 degradation via ubiquitination and the 26S proteasome pathway, thereby controlling the activity of plasma membrane H (+)-ATPases and the acquisition of iron. Therefore, it is thought that IbBT4 might regulate the BR signaling pathway by interacting with BEE2, which results in enhanced drought tolerance in transgenic *Arabidopsis*.

The ABA signaling pathway plays important roles in plant responses to drought stress. Overexpression of *IbARF5* and *IbbZIP1* from sweetpotato enhanced drought tolerance in transgenic *Arabidopsis* through the ABA signaling pathway (Kang et al., 2018, 2019). It has also been reported that H_2_O_2_ plays a role in upstream signaling molecules of the ABA signaling pathway (Leon et al., 2014; Saxena et al., 2016; Phillips and Ludidi, 2017). We also investigated the function of *IbBT4* in the ABA signaling pathway and found that the expression of *IbBT4* was induced by ABA and H_2_O_2_ (Figure 3 and Supplementary Figure S5). However, no difference in seed germination or plant growth on MS media supplemented with ABA was observed between transgenic *Arabidopsis* and WT. Under drought stress, there was no difference in the content of ABA or transcript levels of the ABA biosynthesis related 9-cis-epoxycarotenoid dioxygenase (*NCED*) and ABA DEFICIENT 1 (*ABA1*) genes in the leaves of the transgenic *Arabidopsis* and WT plants (Supplementary Figure S5). These results suggest that *IbBT4* might not be involved in ABA signaling pathway in transgenic *Arabidopsis*.

### *IbBT4* Increases Proline Accumulation and Activates the ROS-Scavenging System

It has been shown that BRs mediate adaptation to abiotic stresses by activating antioxidant machinery, promoting the production of osmoprotectants and fine-tuning stress-responsive transcript machinery (Planas-Riverola et al., 2019). Increases in SOD, CAT, APX, and POD of plants treated with BR indicate that BR act on the antioxidant system and reduce ROS levels, which increase tolerance to the water deficit in cowpea plants (Lima and Lobato, 2017). *TaBZR2* and *TaGST1* modulate drought tolerance in wheat by mediating the crosstalk between BR and ROS-scavenging system (Cui et al., 2019). Overexpression of *BRL3*, a vascular-enriched member of the BR receptor family, triggered proline accumulation and conferred drought stress tolerance to *Arabidopsis* (Fàbregas et al., 2018). Proline is essential for plants responses to abiotic stresses, and proline accumulation can protect plants against ROS damage by altering the activity of antioxidant enzymes (Alia et al., 2001; Khedr et al., 2003; Ozden et al., 2009; Lehmann et al., 2010; Carvalho et al., 2013; Liu et al., 2014). The ROS-scavenging system can protect the structure and function of biomolecules by detoxifying ROS to reduce oxidative damage in plant cells (Melchiorre et al., 2009; Gill and Tuteja, 2010; Liu et al., 2014; Wang et al., 2017).

In this study, we found that the proline content increased and that its key biosynthesis enzyme-encoding genes *AtP5CR* and *AtP5CS* were upregulated (Figures 6E, 7). In addition, in transgenic *Arabidopsis* under drought stress, the SOD activity increased; the ROS-scavenging system genes, including *AtSOD*, *AtGPX*, *AtAPX*, and *AtCAT*, were upregulated; and ROS levels decreased (Figures 6G, 7). These results suggest that overexpression of *IbBT4* increases proline accumulation and activates the ROS-scavenging system, which leads to enhanced drought tolerance in transgenic *Arabidopsis* (Figure 9).

## Conclusion

This study revealed that *IbBT4* positively regulates plant drought tolerance. Under drought stress, *IbBT4* overexpression in *Arabidopsis* significantly enhanced drought tolerance; increased BR and proline contents and SOD activity; decreased ROS and MDA levels; and upregulated genes involved in the BR signaling pathway, proline biosynthesis and the ROS-scavenging system. These results suggest that the *IbBT4* gene provides drought tolerance by enhancing both the BR signaling pathway and proline biosynthesis and further activating the ROS-scavenging system in transgenic *Arabidopsis*.

## Data Availability Statement

The raw data supporting the conclusions of this article will be made available by the authors, without undue reservation, to any qualified researcher.

## Author Contributions

QL and YZ conceived and designed the experiments. YZ and HZu performed the experiments. YZ, HZa, SH, and SG analysed the data. QL, NZ, SX, and ZW contributed to the reagents, materials, and analysis tools. QL and YZ wrote the manuscript. All authors read and approved the final manuscript.

## Conflict of Interest

The authors declare that the research was conducted in the absence of any commercial or financial relationships that could be construed as a potential conflict of interest.

## Footnotes

1 https://blast.ncbi.nlm.nih.gov/Blast.cgi

2 https://www.ncbi.nlm.nih.gov/

## Supplementary Material

FIGURE S1 Promoter of *IbBT4* showing different cis-acting regulatory elements associated with abiotic stress responses.

FIGURE S2 Expression analysis of *IbBT4* indifferent tissues of Xushu55-2. The data are presented as the means ± SEs (n = 3). The different capital letters indicate a significant difference at *P* < 0.01 according to Student’s *t*-test. L: Leaf; S: Stem; HR: Hairy root; FR: Fibrous root; SR: Storage root.

FIGURE S3 Expression analysis of *IbBT4* intransgenic *Arabidopsis* plants. *Atactin* was used as an internal control. The data are presented as the means ± SEs (n = 3). ^∗∗^ Indicates a significant difference from L1 at *P* < 0.01 according to Student’s *t*-test.

FIGURE S4 Transactivation activity assay of *IbBT4* inyeast. **(A)** Transformed yeast cells harbouring different expression vectors were drawn onto SD/-Trp media. pBD (−) and pGAL4 (+) were used as negative and positive controls, respectively. **(B)** Transformed yeast cells harbouring different expression vectors were drawn onto SD/-Trp/-His media supplemented with X-α-Gal. pBD (−) and pGAL4 (+) were used as negative and positive controls, respectively.

FIGURE S5 Analysis of the function of *IbBT4* inthe ABA signalling pathway. **(A)** Expression analysis of *IbBT4* in *in vitro*-grown Xushu55-2 plants after different time points (h) in response to 100 μM ABA. **(B)** Responses of transgenic *Arabidopsis* and WT seeds sown on 1/2 MS media with 0, 0.1, 0.2 and 0.3 μM ABA for 1 week. **(C)** Responses of transgenic *Arabidopsis* and WT seedlings cultured for 2 weeks on 1/2 MS media supplemented with 1 μM ABA. **(D)** ABA content in the leaves of transgenic *Arabidopsis* and WT plants grown for 2 weeks under normal conditions followed by 2 weeks of drought stress and for 4 weeks under normal conditions (control), respectively. **(E–F)** Transcript levels of ABA-related genes in the leaves of transgenic *Arabidopsis* and WT plants grown for 2 weeks under normal conditions followed by 2 weeks of drought stress and for 4 weeks under normal conditions (control), respectively. The data are presented as the means ± SEs (n = 3). ^∗∗^ Indicates a significant difference at *P* < 0.01 according to Student’s *t*-test.

TABLE S1 Primers used in this study.
