# Supplementary material for: The Sweetpotato BTB-TAZ Protein Gene, IbBT4, Enhances Drought Tolerance in Transgenic Arabidopsis
Source: Front Plant Sci. 2020 Jun 23;11:877. doi: 10.3389/fpls.2020.00877 (PMC7324939; doi:10.3389/fpls.2020.00877)
Supplement: FIGURE S1 — Promoter of IbBT4 showing different cis-acting regulatory elements associated with abiotic stress responses. [file Data_Sheet_1.pdf]

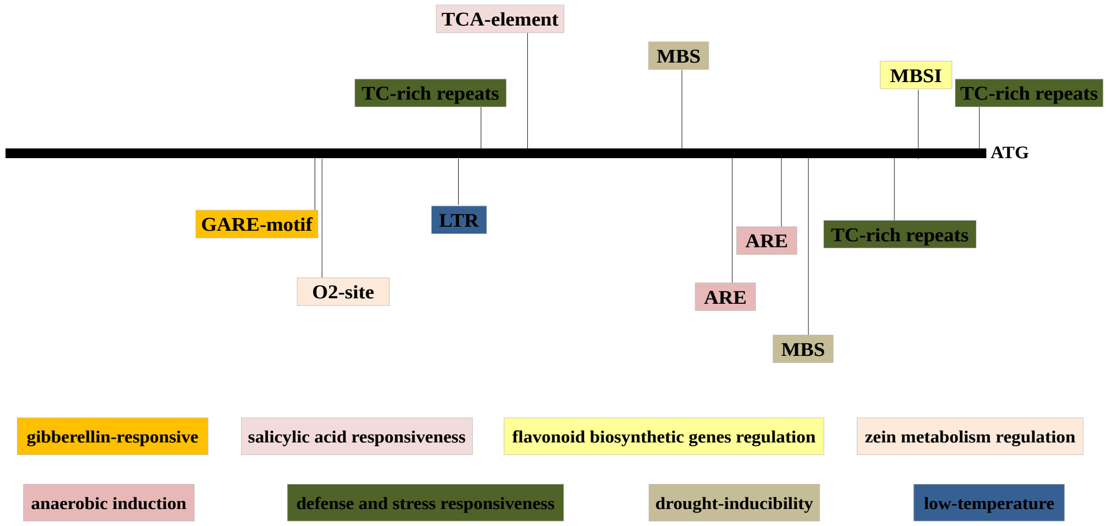

FIGURE S1 | Promoter of *IbBT4* showing different *cis*-acting regulatory elements associated with abiotic stress responses.

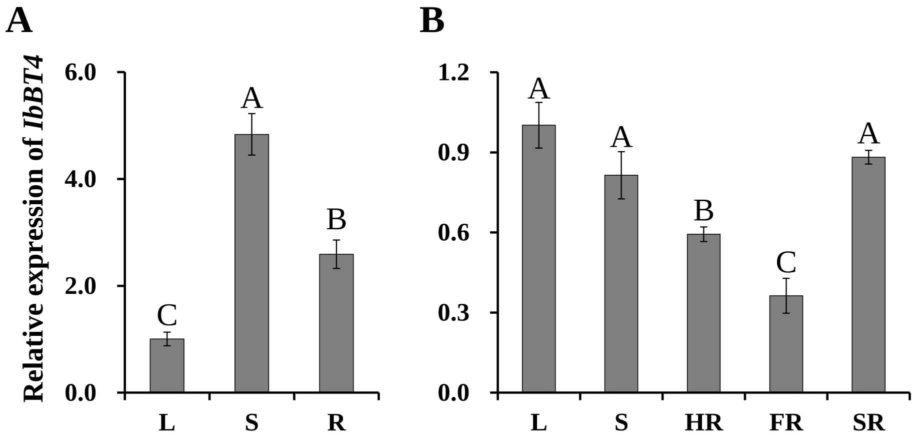

FIGURE S2 | Expression analysis of *IbBT4* in different tissues of Xushu55-2. The data are presented as the means ± SEs (n = 3). The different capital letters indicate a significant difference at P <0.01 according to Student's t-test. L: Leaf; S: Stem; HR: Hairy root; FR: Fibrous root; SR: Storage root.

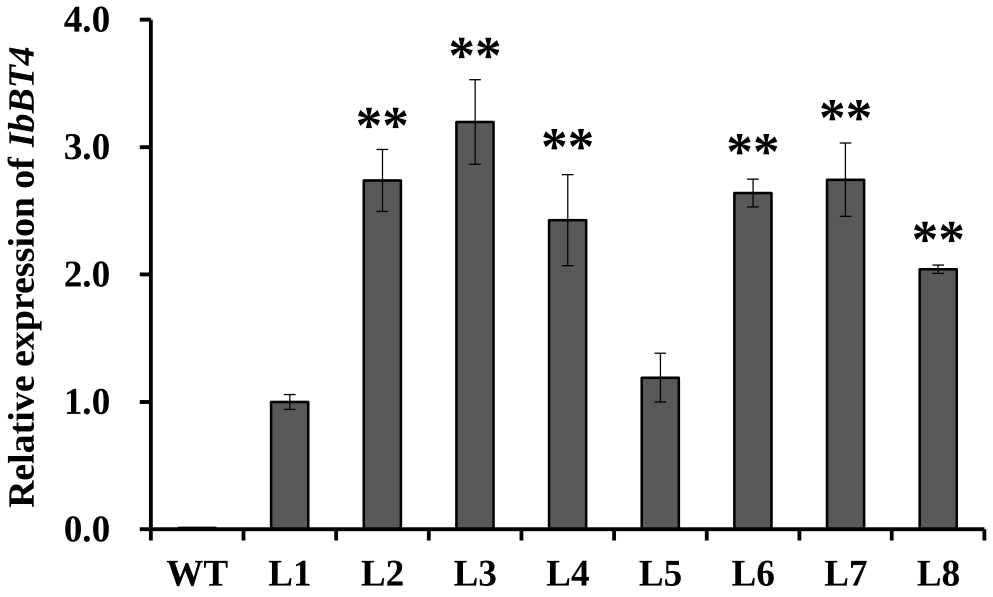

FIGURE S3 | Expression analysis of *IbBT4* in transgenic *Arabidopsis* plants. *Atactin* was used as an internal control. The data are presented as the means ± SEs (n=3). ** indicates a significant difference from L1 at *P*<0.01 according to Student's *t*-test.

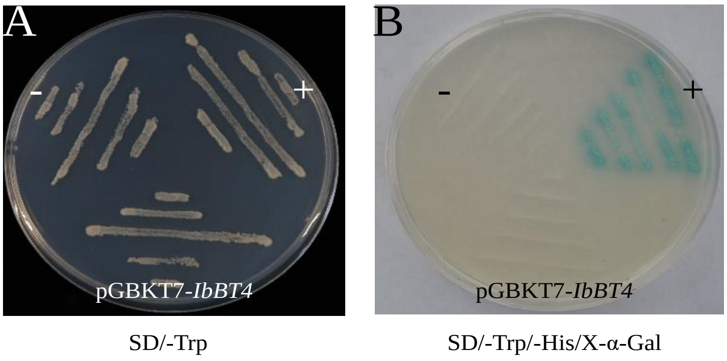

FIGURE S4 | Transactivation activity assay of *IbBT4* in yeast. (**A**) Transformed yeast cells harbouring different expression vectors were drawn onto SD/-Trp media. pBD (-) and pGAL4 (+) were used as negative and positive controls, respectively. (**B**) Transformed yeast cells harbouring different expression vectors were drawn onto SD/-Trp/-His media supplemented with X-α-Gal. pBD (-) and pGAL4 (+) were used as negative and positive controls, respectively.

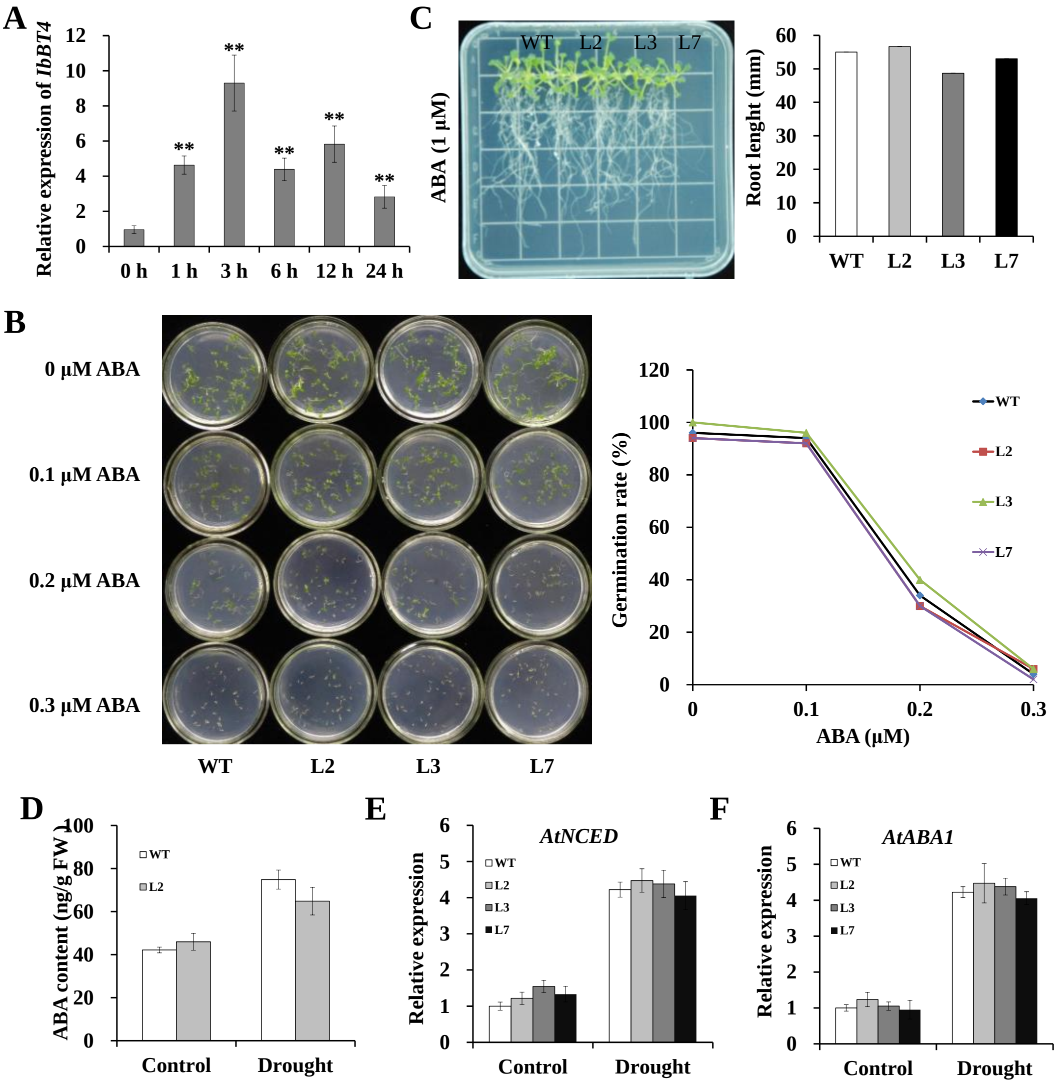

FIGURE S5 | Analysis of the function of *IbBT4* in the ABA signalling pathway. **(A)** Expression analysis of *IbBT4* in *in vitro*-grown Xushu55-2 plants after different time points (h) in response to 100 μM ABA. **(B)** Responses of transgenic *Arabidopsis* and WT seeds sown on 1/2 MS media with 0, 0.1, 0.2 and 0.3 μM ABA for 1 week. **(C)** Responses of transgenic *Arabidopsis* and WT seedlings cultured for 2 weeks on 1/2 MS media supplemented with 1 μM ABA. **(D)** ABA content in the leaves of transgenic *Arabidopsis* and WT plants grown for 2 weeks under normal conditions followed by 2 weeks of drought stress and for 4 weeks under normal conditions (control), respectively. **(E-F)** Transcript levels of ABA-related genes in the leaves of transgenic *Arabidopsis* and WT plants grown for 2 weeks under normal conditions followed by 2 weeks of drought stress and for 4 weeks under normal conditions (control), respectively. The data are presented as the means ± SEs (n = 3). ** indicates a significant difference at P <0.01 according to Student's *t*-test.

Table S1 Primers used in this study

Primer name	Primer sequence (5’-3’)
Primers for 5’/3’ RACE	
5GSP1	CGAAGACACACCGAGAACAC
5GSP2	GCGAACAAAAATGGAGACAG
3GSP1	CGAAAACTTTAGGCAGCAGG
3GSP2	AGTCCGTTTCCTTCACCGAA
Primers for 5’- promoter region	
GW1	TTACTCTGCTCCGACGATGA
GW2	CATCATAGCGGGAAGAATACA
Pro-F	CTCAACTCCCAAGTCCCATC
Pro-R	CTTTCCGATCCTTAAATTTCTGC
Primers for vector construction	
IbBT4-F	ATGGGTAAGCTTTCGGATTC
IbBT4-R	TCATGTTGCTTCAACTGAGAAAAAT
IbBT4-DW-F (*Pac* Ⅰ)	CCTTAATTAAATGGGTAAGCTTTCGGATTC
IbBT4-DW-R (*Asc* Ⅰ)	GGCGCGCCATGTTGCTTCAACTGAGAAAAAT
IbBT4-OS-F(*Xba* Ⅰ)	GCTCTAGAATGGGTAAGCTTTCGGATTC
IbBT4-OS-R(*Pst* Ⅰ)	AACTGCAGTGTTGCTTCAACTGAGAAAAAT
pBD-F-*Nde*I	GGAATTCATGGGTAAGCTTTCGGATTC
pBD-R-*Sal*I	AACTGCAGTCATGTTGCTTCAACTGAGAAAAAT
IbBEE-AD-F(*Nde* Ⅰ)	GGAATTCGAATTCATGCTTCGCTGCGCGC
IbBEE-AD-F(*Bam* H Ⅰ)	CGGGATCCTCACTTTCCCAACCTTGCAGC
AtBEE-AD-F(*Nde* Ⅰ)	GGAATTCGAATTCATGGACTTGTCTGTACTTGATA
AtBEE-AD-F(*Bam* H Ⅰ)	CGGGATCCTTACTTGAGGCTGAAGAAATTGG
CE-IbBEE-F(*Asc* I)	GGCGCGCCATGCTTCGCTGCGCGC
CE-IbBEE-R(*Kpn* I)	GGGGTACCCTTTCCCAACCTTGCAGC
CE-AtBEE-F(*Asc* I)	GGCGCGCCATGGACTTGTCTGTACTTGATAGG
CE-AtBEE-R(*Kpn* I)	GGGGTACCCTTGAGGCTGAAGAAATTGG
NE-IbBT4-F(*Asc* I)	GGCGCGCCATGGGTAAGCTTTCGGATTC
NE-IbBT4-R(*Kpn* I)	GGGGTACCTGTTGCTTCAACTGAGAAAAAT
Primers for transformant identification	
pSuper-1300-F	GACGCCATTTCGCCTTTTCA

pSuper-1300-R	TGAACTTGTGGCCGTTTACGTC
Primers for qRT-PCR	
Ibactin-F	AGCAGCATGAAGATTAAGGTTGTAGCAC
Ibactin-R	TGGAAAATTAGAAGCACTTCCTGTGAAC
IbBT4-F	CCGATTATGAAAGCCATGTTGAG
IbBT4-R	TACGAATGCGACAGCACCAGTAA
Atactin-F	GCACCCTGTTCTTCTTACCGA
Atactin-R	AGTAAGGTCACGTCCAGCAAGG
AtDWF4-F	CCGTACACCGCCACAA
AtDWF4-R	GAATCTATTAAGTCCAGCATCAG
AtCPD-F	GCTGATCGGAGCTTACAAAAC
AtCPD-R	AAATCGTCGGTTCACCAAAA
AtDET2-F	CACCAACCGCCGTCCTT
AtDET2-R	CGGTGGAGATACGGTGGGAC
AtROT3-F	AACTTCATCGCTTGTGGTTATT
AtROT3-R	TTGGTGTCCCTATTATGTTCGT
AtCYP90D1-F	TTTATCATCATCGTCATCTTCA
AtCYP90D1-R	TTTGGTCCGTGACTCTGG
AtBR6ox1-F	AAACCAAAGACTCCGATACGG
AtBR6ox1-R	CGATTGTGGGTAACCAGGAA
AtBR6ox2-F	ACCAAAGACTAAGATATGGGAGT
AtBR6ox2-R	AAGCATAGATTGCGGGTAA
AtBRI1-F	CTCTCCTGTCTCTCACCGGA
AtBRI1-R	GCACCGGAGATTGAATTCGC
AtBIN2-F	AGATGCCTGCTGCTGTAGTTG
AtBIN2-R	CCACGGTTTCTCCAGTCTCC
AtBZR1-F	ATGGTGGCATTCCTTCTTCTC
AtBZR1-R	GCAACGGTTTCGGGTTCTT
AtBES1-F	CCCAAACCATTGCCTACTTG
AtBES1-F	GGTGCAGACACCGCATAAAA
AtBR6ox1-F	AAACCAAAGACTCCGATACGG
AtBR6ox1-R	CGATTGTGGGTAACCAGGAA
AtBR6ox2-F	ACCAAAGACTAAGATATGGGAGT

AtBR6ox2-R	AAGCATAGATTGCGGGTAA
AtSOD-F	ATGAGAAGTTCTATGAAGAG
AtSOD-R	GTCTTTATGTAATCTGGT
AtGPX-F	ATGGCGACGAAGGAACCAG
AtGPX-R	ATCGCCGAAGATTCCCCATTT
AtPOD-F	TCCGGGAGCACACCATTGG
AtPOD-R	TGGTCGGAATTCAACAG
AtCAT-F	GCAACTACCCCCGAGTGGAAA
AtCAT-R	TGTTCAGAACCAAGCGACCA
AtNCED-F	AGAAGCAGGGCAAATAAACAAG
AtNCED-R	CCGTCGCCGTACCTAAACTC
AtABA1-F	TACTTGGGGTAAAGGGCGTG
AtABA1-R	CAAAGCACCCTGCAATAACT
IbBEE-F	GAGGAAGATAAGAAATGGGAAGGAGA
IbBEE-R	ATGGCTGTCGGTGGCTTGG
